# From beauty to burden: mapping the health and psychosocial impacts of fragrances and cosmetic products use in the UAE

**DOI:** 10.3389/ftox.2026.1758512

**Published:** 2026-04-15

**Authors:** Sharifa AlBlooshi, Mariam Al Ali, Dana N. Abdelrahim, Suheir Awadalla

**Affiliations:** 1 Department of Health Sciences, College of Natural and Health Sciences, Zayed University, Dubai, United Arab Emirates; 2 Research Institute for Medical and Health Sciences, University of Sharjah, Sharjah, United Arab Emirates; 3 Department of Psychology, College of Natural and Health Sciences, Zayed University, Dubai, United Arab Emirates

**Keywords:** adverse effects, cosmetics, dermatitis, mental health, perfume, respiratory tract diseases, United Arab Emirates, women

## Abstract

**Background:**

The dynamic beauty care sector in the Middle East is on an upward trend in the UAE; however, concerns persist about the side effects of this industry, attributed to chemical exposure, which is believed to contribute to skin problems and other health issues. In the UAE, there is a lack of data on consumer usage, awareness, and the health effects associated with it.

**Methods:**

This study presents the results of an online survey administered to 461 female subjects in the United Arab Emirates as part of a cross-sectional study. The survey consisted of 55 questions on product use, knowledge, attitudes, safety measures, and potential adverse health effects. Descriptive statistics and logistic regression analysis were applied to identify predictors of adverse reactions.

**Results:**

Among the 461 participants, most were aged 18–29 years (55.7%) and university educated (68.3%). The application of scent and makeup was almost widespread, as 91.5% of users applied scented products daily, and 100% of users mentioned cosmetics, 36.4% of respondents reported health problems caused by the use of these products, with the most common issues being persistent respiratory problems (16.3%), headaches (15.8%), and skin irritation (11.9%). Knowledge of fragrance safety was moderate, with only 43.8% recognizing that fragrances contain complex chemical mixtures. The study demonstrated daily cosmetic use in 75.1% of individuals who frequently perform allergy tests, and limited safety practices in 6.7% of these individuals. While allergy testing was protective, logistic regression analysis revealed that younger age, higher education, a history of allergies, and frequent product use were predictors of adverse events. This has led to the association of widespread use of fragrances and cosmetics amongst women in the UAE with major respiratory and dermatological problems.

## 1 Introduction

The beauty sector was valued at $532 billion by the end of 2020. In 2025, the UAE’s beauty sector is projected to reach $3 billion. Therefore, it is crucial for daily living and personal hygiene. Cosmetic products and perfumes play a significant role in consumers’ daily lives, serving as a means of self-expression and personal beauty. On the other hand, as more data become available, cosmetics have also been found to pose a health threat due to the presence of hazardous chemicals, heavy metal use, and endocrine disruptors, all of which may have adverse health implications.

The long-term use of cosmetics can be harmful, causing cancer, reproductive problems, and detectable adverse effects, as well as health issues such as skin irritation, allergic responses, and respiratory problems. Cosmetics can contain heavy metals such as lead, mercury, and chromium, which pose toxicological risks including carcinogenesis, neurotoxicity, and developmental toxicity. They are often found in makeup and tattoo ink products, as well as in other sources. Continued client exposure is also enabled in several countries due to weak compliance with regulatory limits, despite strong regulations (Alblooshi, 2025; Alosyli, 2024; Athar, 2020; Browwska and Brzoska, 2025; Huang, 2018; Air Quality Energy and Health, 2010).

A study has shown that fragrance use influences spontaneous brain activity and cognitive functions (Sowndhararajan and Kim, 2016). Despite these alarming results, the long-term health effects remain unknown, and consumers are not informed about them. Additionally, the majority of available research currently focuses on Western populations, leaving a knowledge gap regarding the consequences peculiar to a specific region, particularly in the UAE.

The beauty industry has flourished over the past decade, with fragrances and cosmetics playing a key role in every consumer’s routine. A survey conducted by Klaschka (2020) found that a significant number of survey respondents were troubled by fragrance usage. As the beauty industry thrives on a burgeoning consumer market driven by new desires, questions have been raised about whether the use of cosmetics and fragrances poses a health risk. Dangerous additives found in cosmetics and fragrances have been associated with many diseases, such as allergies, cancer, challenges for expectant mothers as they strive to conceive, and even an unspecified host of other quite undesirable conditions. As a result, evaluating the potential safety risks to human health associated with cosmetics and perfumes becomes crucial (Mohammed, 2023; Naveed, 2014; Odencrantz and Offenberg, 2020; Sealey, 2016; Steinemann, 2018).

It has been demonstrated that 80.1% of females use at least 1 cosmetic product, such as skincare, lotions, lipsticks, and eye makeup (Getachew and Tewelde, 2018). An observational cross-sectional study in Saudi Arabia showed that 50.6% of the participants had unpleasant reactions after using cosmetics (Lucca et al., 2020). Panico found that 52.3% of the products contained fragrances such as limonene and linalool, preservatives like phenoxyethanol showed a rate of 60%, and other chemicals of concern, which were detected in 58% of the products, like polyethylene glycols and petrolatum (Panico et al., 2019). Parabens, a type of preservative, are classified as endocrine disruptors as they chemically imitate the estrogenic activity, which may lead to various health outcomes, including breast, ovarian, and testicular cancer. Exposure to the other chemicals found in the products can cause neuronal delay, congenital malformations, and fertility deficiency in men. Shaaban and Alhaari (2020) found that 16.1% of people experience adverse health problems associated with cosmetic use. Although these ingredients in beauty products are produced in small quantities, continuous exposure to large doses over time could have detrimental effects on consumer health (Shaaban and Alhajri, 2020).

This study was designed to (1) investigate the pattern of cosmetic and fragrance usage among women in the UAE, (2) evaluate the self-reported health and psychosocial consequences of the usage of cosmetics and fragrances, and (3) explore the predictors of adverse reactions. To the best of our knowledge, this study is among the first in the UAE to investigate the physical and psychosocial consequences of cosmetic and fragrance use in a large sample.

### 1.1 Study design and setting

A quantitative cross-sectional design was employed to assess the health problems associated with the use of cosmetics and fragrances among females in the UAE. The data were gathered from an online survey on social media and via email. Data collection was conducted between February and May 2025. A total of 503 responses were obtained; 42 men’s questionnaires were excluded from the survey as they were less representative of the sex distribution in this population. Male responses were excluded to focus on the use of cosmetics and fragrances among women, who are the main target audience for these products in the UAE society. The results may not be generalized for the male population. A confidence sampling using the snowball method was implemented. The survey was designed using Google Forms, comprising three sections with a total of 55 items. These sections were as follows: Section 1 demographics information about oneself, Section 2 the effects of cosmetics use on health, and Section 3 fragrance exposure-related health harms suffered by people taking any kind of product that has an aroma about it, at all times. A pilot study was conducted on 20 students from Zayed University to test the survey before the data collection. The sample size was calculated using the single-population proportion formula for a 50% expected prevalence (to maximize variability), a 95% confidence level, and a 5% margin of error. The minimum required sample size was 385 participants. The target sample size was 424 participants, adjusted for a potential 10% non-response rate. A total of 461 participants were finally included, exceeding the minimum required sample size and thus enhancing the precision of the estimates.

### 1.2 Questionnaire development

A structured questionnaire was developed by combining and revising items from three studies on fragrance exposure, cosmetic use, and associated health consequences (Getachew and Tewelde, 2018; Klaschka, 2020; Lucca et al., 2020). The structured questionnaire was tested with academic experts at Zayed University to gather their suggestions, assess comprehensibility, and evaluate practical applicability. The questionnaire finally consisted of 55 items, under eight headings: (1) sociodemographic characteristics (5 items: sex, age, education, marital status, occupation); (2) fragrance exposure (6 items); (3) health problems related to exposure (10 items); (4) knowledge, attitudes and practices (8 items); (5) cosmetic use patterns (10 items); (6) safety practice (3 items); (7) adverse reactions (6 items); and (8) mental health signals (7 items).

### 1.3 Ethics approval

On 12 February 2025, the Zayed University Research Ethics Committee granted ethical approval for this study (Approval No. ZU25_002_F). All participants provided informed consent electronically before participation, as part of the online survey.

### 1.4 Data processing and statistical analysis

Data analysis was conducted using SPSS software version 29. Descriptive statistics, specifically frequencies, were used to provide an overview of the categorical responses. Microsoft Excel was used to visualize the results. A logistic regression model was used to assess the determinants of cosmetic-related adverse reactions among the respondents. All significant variables from the bivariable logistic regression were entered into the multivariable logistic regression. The 95% CI was used to show the strength of association and statistical significance of predictors. A p-value of less than 0.05 was set to assess the significance.

## 2 Results

### 2.1 Socioemographic characteristics

The sociodemographic characteristics of the participants are presented in Table 1. The ages of the 461 participants were as follows: 55.7% were 18–29 years old, 23.0% were 30–40 years old, 14.3% were 41–50 years old, and 6.9% were over 50 years old.

**TABLE 1:** Sociodemographic characteristics (n = 461).

Variable	Category	Frequency	Percent
Age group	18–29 years	257	55.7
	30–40 years	106	23
	41–50 years	66	14.3
	Above 50 years	32	6.9
Education	Lower than secondary	7	1.5
	Secondary	98	21.3
	University	315	68.3
	Postgraduate	41	8.9
Occupation	Student	191	41.4
	Employed	136	29.5
	Unemployed	114	24.7
	Retired	20	4.3
Marital status	Single	250	54.2
	Married	194	42.1
	Divorced	13	2.8
	Widowed	4	0.9

A minor proportion (8.9%) of respondents held postgraduate qualifications, as compared to the largest segment (68.3%) who were college graduates. Regarding occupation, students represented the largest group (41.4%), followed by employed individuals (29.5%), unemployed participants (24.7%), and retired individuals (4.3%). In terms of marital status, over half of the sample were single (54.2%), and 42.1% were married, with only a small proportion divorced (2.8%) or widowed (0.9%).

### 2.2 Exposure to fragranced products

**FIGURE 1:**
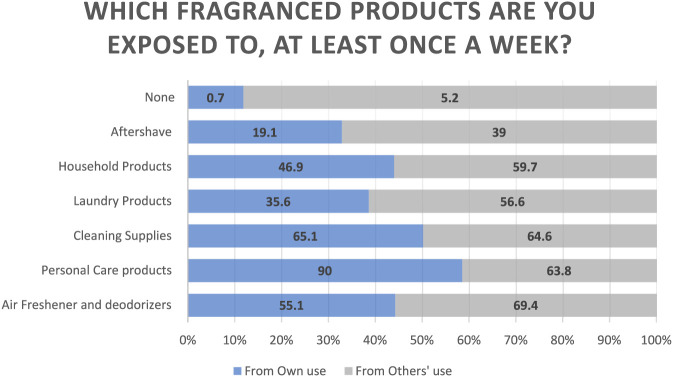
Exposure to fragrance (from own use and from others’ use).

Table 2 summarizes weekly exposure to fragranced products from participants’ own use and from others. Exposure to fragranced products at least once a week was reported from both personal use and others’ use, as shown in Figure 1. More than half (55.1%) reported personal use of air fresheners or deodorizers, while exposure from others was higher (69.4%). The majority used personal care products weekly (90.0%) and were exposed to them through others’ use (63.8%). Cleaning supplies (e.g., all-purpose cleaners, disinfectants, bleach products, and dishwashing liquids) were another major source, with 65.1% using them personally and 64.6% reporting secondary exposure. Laundry products were less commonly used for personal purposes (35.6%), but were widely encountered through others (56.6%). Similarly, household products such as candles or air fresheners were used by 46.9% of respondents, and were also encountered through others’ use in 59.7%. Aftershave exposure was less frequent (19.1% own, 39.0% from others), reflecting either personal use or exposure from others. Only 0.7% reported no personal exposure to fragrance, and 5.2% reported no exposure through others’ use. Fragrance use was nearly universal, with 91.5% using such products daily, and most used 1–5 products per day (87.4%). About 35.1% applied fragrance twice daily, while 26.9% applied it once daily. Regarding spending, 38.4% reported spending more than 500 AED per month, and 34.9% spent between 200 and 500 AED, highlighting both high frequency and significant financial investment in fragranced products.

**TABLE 2:** Fragrance exposure (n = 461) [n (%)].

Variable		From Own use	From Others’ use
Exposure to fragranced products (at least once a week) MAQ^a^	Air freshener and deodorizers (e.g., sprays, solids, oils, disks)	254 (55.1)	320 (69.4)
	Personal care products (e.g., soaps, hand sanitizer, lotions, deodorant, sunscreen, shampoos)	415 (90.0)	294 (63.8)
	Cleaning supplies (e.g., all-purpose cleaners, disinfectants, and dishwashing soap)	300 (65.1)	298 (64.6)
	Laundry products (e.g., detergents, fabric softeners)	164 (35.6)	261 (56.6)
	Household products (e.g., scented candles, toilet paper, trash bags, baby products)	216 (46.9)	275 (59.7)
	Aftershave (e.g., lotion, cologne)	88 (19.1)	180 (39.0)
	None	3 (0.7)	24 (5.2)
Frequency of using fragrance	Daily	422 (91.5)	
	Weekly	24 (5.2)	
	Monthly	4 (0.9)	
	For special occasions	9 (2.0)	
	Rarely	1 (0.2)	
	Never	1 (0.2)	
Number of fragrance products used daily	1–2 times	196 (42.5)	
	3–5 times	207 (44.9)	
	6–10 times	30 (6.5)	
	More than 10	22 (4.8)	
	Not applicable	6 (1.3)	
Frequency of fragrances usually used in a day	Once	124 (26.9)	
	Twice	162 (35.1)	
	Three times	121 (26.2)	
	Four times or more	50 (10.8)	
	Not applicable	4 (0.9)	
Money spent on fragrance monthly in the UAE dirham	Less than 100 AED	38 (8.2)	
	100–200 AED	64 (13.9)	
	200–500 AED	161 (34.9)	
	More than 500 AED	177 (38.4)	
	Not applicable	21 (4.6)	

^a^ MAQ: multiple answer question, which further means that the total cannot be summed up toa hundred.

### 2.3 Health problems experienced by participants

**FIGURE 2:**
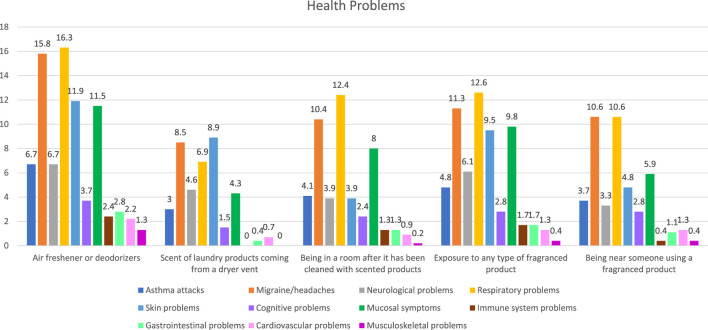
Health problems among participants.

As shown in Table 3, participants (n = 461) reported health problems from various sources. Specifically, 37% reported health problems resulting from air fresheners or deodorizers, while 31% reported general exposure to any fragranced product. Rooms cleaned with aromatic products produced health problems for 28% of respondents, and standing near someone using a scented product for 24%. As for the smell of laundry products, it caused 23% of participants to produce symptoms of health problems. Amongst all the sorts of exposure, most frequently reported symptoms matched closely with respiratory problems and anxiety. Respiratory problems affected up to 16% of participants, and headaches resulted in injuries in 15%. This was dialed up several notches, especially after being near an air freshener. One of the subsequent sets of symptoms concerned skin problems (12%), mucosal irritation (e.g., watery eyes or nasal congestion) at 11%, and neurological problems such as “the vertigo” or fainting at 6%. Rarely, complaints turned to cognitive difficulties, immune system problems, gastric troubles, intestinal complaints, and heart diseases. Each of these sections had fewer than 4% of its voter representatives. It is noteworthy that people are highly variable when using fragranced products (as seen across 61.2%–77.2% of cases, depending on the type of exposure). Health problems are also presented in Figure 2.

**TABLE 3:** Which of the following health problems do you experience when exposed to? (n = 461) [n (%)].

Health problems MAQ		Exposed to?				
		Air freshener or deodorizers	Scent of laundry products coming from a dryer vent	Being in a room after it has been cleaned with scented products	Exposure to any type of fragranced product	Being near someone using a fragranced product
Do you experience any health problems from the following?		**173 (37.5)**	**106 (23.0)**	**131 (28.4)**	**146 (31.7)**	**111 (24.1)**
Which of the following health problems do you experience from the product? MAQ	Asthma attacks	31 (6.7)	14 (3.0)	19 (4.1)	22 (4.8)	17 (3.7)
	Migraine/headaches	73 (15.8)	39 (8.5)	48 (10.4)	52 (11.3)	49 (10.6)
	Neurological problems (e.g., dizziness, seizures, head pain, fainting)	31 (6.7)	21 (4.6)	18 (3.9)	28 (6.1)	15 (3.3)
	Respiratory problems (e.g., difficulty breathing, coughing)	75 (16.3)	32 (6.9)	57 (12.4)	58 (12.6)	49 (10.6)
	Skin problems (e.g., rashes, hives, red skin, tingling skin, dermatitis)	55 (11.9)	41 (8.9)	18 (3.9)	44 (9.5)	22 (4.8)
	Cognitive problems (e.g., difficulties thinking, concentrating, or remembering)	17 (3.7)	7 (1.5)	11 (2.4)	13 (2.8)	13 (2.8)
	Mucosal symptoms (e.g., watery or red eyes, nasal congestion, sneezing)	53 (11.5)	20 (4.3)	37 (8.0)	45 (9.8)	27 (5.9)
	Immune system problems (e.g., fever, fatigue)	11 (2.4)	0 (0.0)	6 (1.3)	8 (1.7)	2 (0.4)
	Gastrointestinal problems (e.g., nausea, bloating, cramping, diarrhea)	13 (2.8)	2 (0.4)	6 (1.3)	8 (1.7)	5 (1.1)
	Cardiovascular problems (e.g., fast or irregular heartbeat, chest discomfort)	10 (2.2)	3 (0.7)	4 (0.9)	6 (1.3)	6 (1.3)
	Musculoskeletal problems (e.g., muscle or joint pain, cramps, weakness)	6 (1.3)	0 (0.0)	1 (0.2)	2 (0.4)	2 (0.4)
	Not applicable	282 (61.2)	356 (77.2)	329 (71.4)	315 (68.3)	351 (76.1)

Bold values indicate the most commonly reported health problems experienced by respondents (n = 461). Values are presented as n (%). Respondents could report multiple health problems for each type of exposure; therefore, percentages may sum to more than 100%.

### 2.4 Participants’ knowledge, attitudes, and practices (KAP) regarding fragranced products

KAP (knowledge, attitudes, and practices) regarding aromatized products are outlined in Table 4. The majority of participants were unaware that most fragrances in products are composed of complex mixtures of chemicals (43.8%) and that only about one-tenth (11.9%) knew that there is no complete disclosure of fragrance chemicals on labels or safety sheets. The level of awareness regarding the harms caused by scented products was inconsistent: 30.2% of participants knew that using these products might result in the release of hazardous air pollutants, while only 19.3% agreed that even so-called natural or organic scent products may release such pollutants. Almost half (47.5%) stated they prefer products with natural fragrance, and 23.0% were completely unaware of the subject. The survey revealed that 74.8% of participants reported using scented products, such as perfumes or deodorants, to enhance their attractiveness, which was the most frequently expressed attitude or practice. Fewer participants actively sought chemical information: 42.7% read product references, and 55.3% indicated that chemical information influences their purchasing decisions.

**TABLE 4:** Knowledge, Attitude, Practice towards fragrances (KAP) (n = 461) [n (%)].

Questions		Yes	No
Knowledge Which of the following do you think is (are) correct concerning fragrances? MAQ	Fragrance in a product is typically a chemical mixture of several dozen to several hundred chemicals	202 (43.8)	259 (56.2)
	Fragrance chemicals do not need to be fully disclosed on the product label or material safety data sheet.	55 (11.9)	406 (88.1)
	Fragranced products typically emit hazardous air pollutants such as formaldehyde	139 (30.2)	322 (69.8)
	Even so-called natural, green, and organic fragranced products typically emit hazardous air pollutants	89 (19.3)	372 (80.7)
	Products with natural fragrance ingredients are healthier than products with synthetic fragrance ingredients	219 (47.5)	242 (52.5)
	None	106 (23.0)	355 (77.0)
Attitude and practice	Do you use scented products, such as perfume or deodorant, to feel more attractive?	345 (74.8)	116 (25.2)
	Do you read the references to the products you use to get information about the chemicals/ingredients they contain?	197 (42.7)	264 (57.3)
	Does the information about a particular chemical/ingredient in a product affect your purchasing decision?	255 (55.3)	206 (44.7)
	Do you believe that products with natural ingredients are healthier than products with synthetic ingredients?	379 (82.2)	82 (17.8)
	Do you prefer fragrance-free products when they are available?	292 (63.3)	169 (36.7)
	Has a doctor or healthcare professional ever told you that you have autism or autism spectrum disorder?	38 (8.2)	423 (91.8)
	Has a doctor or healthcare professional ever told you that you have asthma or an asthma-like condition?	76 (16.5)	385 (83.5)

On the other hand, a very large percentage (82.2%) of the participants were convinced that products with natural ingredients are healthier, and 63.3% preferred fragrance-free products. Reports on the doctor’s opinion were quite rare; 8.2% were informed by a doctor about autism, and 16.5% were comorbid with asthma or a similar condition.

### 2.5 Participants’ cosmetic utilization patterns

Table 5 shows how the participants used cosmetics. All participants (100%) have at least one cosmetic product. The highest rate was found in skincare or hair care (89.4%), followed directly by personal care products (83.7%). The makeup was increasingly popular at an 82.4% rate. Perfumes (75.9%) and nail care products (66.2%) were also very common, whereas traditional/herbal cosmetics products were used by about half (50.8%) of the participants. Most respondents use cosmetics once a day (75.1%), with 46.9% of the population applying three to five products daily and 24.1% applying one or two. Regarding usage frequency during the day, 43.8% applied cosmetics twice daily, and 32.8% applied them once. Moreover, sharing cosmetics was not a frequent practice: 34.1% of users rarely shared their products, and 22.6% never shared theirs at all. The purchases were highly concentrated in cosmetic shops (87.9%) and pharmacies (64.6%), with more than half (56.6%) purchased from online stores and supermarkets (44.7%). In the process of choosing, the criteria laid down placed the highest marks on quality (69.4%), followed by brand reputation (58.4%) and recommendations (56.6%), while only 13.4% considered advertisements as a decision-making factor. The prime places to store cosmetics were room cabinets (79.8%) and bathrooms (53.6%). Monthly expenditure was considerable: 37.7% of respondents spent between 200 and 500 AED, and 35.4% spent over 500 AED.

**TABLE 5:** Utilization pattern of cosmetics (n = 461) [n (%)].

Question/Category		[N (%)]
Do you use any cosmetics (e.g., creams, lotions, shampoos, hair dyes, lipsticks, foundations, deodorants, shaving products, nair care, and traditional cosmetics like eggs)?		461 (100.0)
What categories of cosmetic products do you use? MAQ	Skincare (e.g., creams, lotions, make-up remover)	412 (89.4)
	Haircare (e.g., shampoos, hair dyes, conditioners, hair spray)	407 (88.3)
	Makeup (e.g., lipsticks, foundations, eyebrow pencil, loose powder, eyeliner)	380 (82.4)
	Perfumes	350 (75.9)
	Personal care (e.g., deodorants, shaving products, toothpaste, soap)	386 (83.7)
	Nail care (e.g., nail polish, nail polish remover)	305 (66.2)
	Traditional/herbal cosmetics (e.g., henna, mud masks, egg, honey)	234 (50.8)
	Not applicable	2 (0.4)
How frequently do you use these cosmetics?	Daily	346 (75.1)
	Weekly	72 (15.6)
	Monthly	10 (2.2)
	Special occasions	17 (3.7)
	Rarely	11 (2.4)
	Never	5 (1.1)
How many cosmetic products do you use daily?	1–2 times	111 (24.1)
	3–5 times	216 (46.9)
	6–10 times	81 (17.6)
	More than 10	47 (10.2)
	Not applicable	6 (1.3)
How frequently do you usually use cosmetics in a day?	Once	151 (32.8)
	Twice	202 (43.8)
	Three times	63 (13.7)
	Four times or more	38 (8.2)
	Not applicable	7 (1.5)
Do you share cosmetics with others (e.g., friends, family members)?	Frequently	27 (5.9)
	Often	42 (9.1)
	Sometimes	131 (28.4)
	Rarely	157 (34.1)
	Never	104 (22.6)
Where do you purchase cosmetics from? MAQ	Pharmacy	298 (64.6)
	Supermarkets/hypermarkets	206 (44.7)
	Cosmetic shops	405 (87.9)
	Online stores	261 (56.6)
What is your criterion for selecting cosmetic products? MAQ	Brand reputation	269 (58.4)
	Recommendations from others	261 (56.6)
	Advertisements	62 (13.4)
	Price/Cost	160 (34.7)
	Quality (e.g., ingredients, safety certifications)	320 (69.4)
Where do you store your cosmetics? MAQ	Room cabinets	368 (79.8)
	Bathroom	247 (53.6)
	Car	45 (9.8)
	Handbags	150 (32.5)
	Fridge	71 (15.4)
How much do you spend on cosmetics per month in the UAE dirham?	Less than 100 AED	35 (7.6)
	100–200 AED	89 (19.3)
	200–500 AED	174 (37.7)
	More than 500 AED	163 (35.4)

### 2.6 The safety practices of participants regarding cosmetic use

Table 6 provides insight into various aspects of cosmetic use among the participants. Approximately 21.7% of the participants indicated that they always read the labels or instructions prior to applying cosmetics, 15.2% did so very regularly, 33.0% occasionally, 19.3% hardly ever, and the remaining 10.8% never did. Checking the expiry date was a more commonly applied practice, with 34.9% of people doing it frequently, followed by 19.3% and 21.7% doing so often and sometimes, respectively. Meanwhile, 16.9% and 7.2% did it only rarely and never, respectively. On the contrary, conducting allergy tests prior to the introduction of new products was a rare practice among the respondents: only 6.7% very often did it, 8.7% did it frequently, 23.6% did it sometimes, 21.0% did it hardly ever, and the largest portion, 39.9%, was composed of those who completely abstained from conducting any allergy tests. Therefore, it was observed that, despite participants displaying some level of caution when reading labels and checking expiry dates, they completely ignored the pre-use allergy testing.

**TABLE 6:** Safety practices (n = 461) [n (%)].

Question		Total population [n (%)]
Do you read the label or instructions before using cosmetics?	Frequently	100 (21.7)
	Often	70 (15.2)
	Smetimes	152 (33.0)
	Rarely	89 (19.3)
	Never	50 (10.8)
Do you check the expiry date of cosmetics?	Frequently	161 (34.9)
	Often	89 (19.3)
	Sometimes	100 (21.7)
	Rarely	78 (16.9)
	Never	33 (7.2)
Do you perform allergy tests before using new products?	Frequently	31 (6.7)
	Often	40 (8.7)
	Sometimes	109 (23.6)
	Rarely	97 (21.0)
	Never	184 (39.9)

### 2.7 The prevalence and characteristics of adverse reactions to cosmetics among the participants

**FIGURE 3:**
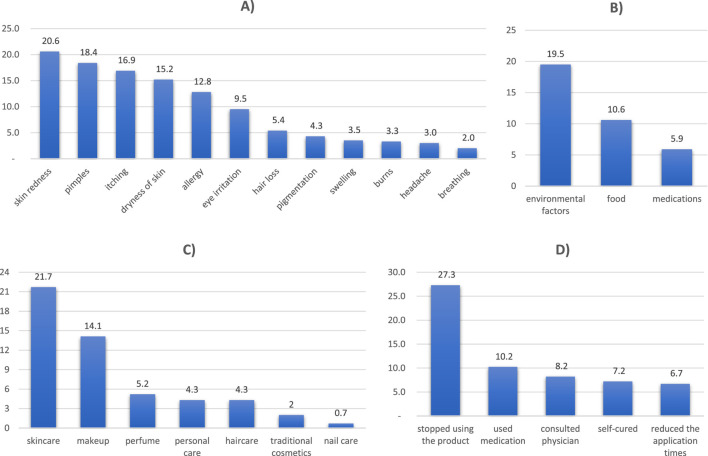
Adverse reaction symptoms, product caused, and how the reaction was managed. **(A)** Symptoms of adverse reaction. **(B)** Types of Allergy. **(C)** Product caused the adverse reaction. **(D)** Management of the adverse reaction.

According to Table 7, the overall prevalence of adverse reactions to cosmetics was 36.4% within the past 2 years, while 63.6% did not experience any adverse reactions. The most common symptoms reported in these reactions were skin redness (20.6%), pimples (18.4%), itching (16.9%), and dryness of skin (15.2%). Less commonly reported symptoms included eye irritation (9.5%), hair loss (5.4%), pigmentation (4.3%), localized swelling (3.5%), burns (3.3%), headache (3.0%), and breathing difficulties (2.0%). More than half of the participants (63.3%) stated that such specific symptoms did not apply to them personally. Skincare products were the most frequently named cause for adverse reactions, accounting for 21.7% of cases, followed by makeup at 14.1%, perfumes accounted for 5.2%, while haircare and personal care products each accounted for 3%. Traditional cosmetics made up just 2%. Additionally, 62.7% of respondents stated that the product type was not applicable. For the treatment, stopping the product was the most frequently mentioned strategy for managing adverse reactions (27.3%), medication use took second place at (10.2%), while seeing a doctor was third (8% 22). Reducing the moot frequency of usage was followed by spontaneously curing the situation at 6%–7%, yet 63.1% reported it as not applicable. In terms of allergies, 46.6% of respondents had a family history, 19.5% experienced personal reactions to environmental factors, 10.6% were allergic to food, and 25.29% had drug reactions; while 64.0% claimed no known allergies. Adverse reactions are also illustrated in Figure 3, along with explanations of both symptoms and their causes.

**TABLE 7:** The Adverse reaction (n = 461) [n (%)].

Question		Yes	No
Have you ever experienced any adverse reactions to cosmetics in the past 2 years?		168 (36.4)	293 (63.6)
If yes, specify the symptoms	Skin redness	95 (20.6)	366 (79.4)
	Itching	78 (16.9)	383 (83.1)
	Dryness of skin	70 (15.2)	391 (84.8)
	Allergy	59 (12.8)	402 (87.2)
	Pimples	85 (18.4)	376 (81.6)
	Eye irritation	44 (9.5)	417 (90.5)
	Swelling	16 (3.5)	445 (96.5)
	Hair loss	25 (5.4)	436 (94.6)
	Burns	15 (3.3)	446 (96.7)
	Breathing	9 (2.0)	452 (98.0)
	Pigmentation	20 (4.3)	441 (95.7)
	Headache	14 (3.0)	447 (97.0)
	Not applicable	292 (63.3)	169 (36.7)
Which products caused these reactions?	Skincare	100 (21.7)	361 (78.3)
	Haircare	20 (4.3)	441 (95.7)
	Makeup	65 (14.1)	396 (85.9)
	Personal care	20 (4.3)	441 (95.7)
	Traditional cosmetics	9 (2.0)	452 (98.0)
	Perfume	24 (5.2)	437 (94.8)
	Nail care	3 (0.7)	458 (99.3)
	Not applicable	289 (62.7)	172 (37.3)
How did you manage the reaction?	Stopped using the product	126 (27.3)	335 (72.7)
	Consulted physician	38 (8.2)	423 (91.8)
	Reduced the application times	31 (6.7)	430 (93.3)
	Used medication	47 (10.2)	414 (89.8)
	Self-cured	33 (7.2)	428 (92.8)
	Not applicable	291 (63.1)	170 (36.9)
Do any of your family members have a history of allergies?		215 (46.6)	246 (53.4)
Do you have any allergies to	Medications	27 (5.9)	​
	Food	49 (10.6)	​
	Environmental factors	90 (19.5)	​
	No known allergies	295 (64.0)	​

### 2.8 Participants’ perceptions of mental health and wellbeing related to fragrance and cosmetic use

Table 8 presents participants’ perceptions of mental health, well-being, stress, and self-perception regarding the use of fragrances and cosmetics. The majority of participants reported feeling relaxed and happy (42.5%) or more confident (26.9%) after using fragrances or cosmetics, compared to feeling “neutral” (19.3%), while far fewer expressed other feelings. When participants were asked about improvements in mood, they tended to report moderate (27.1%) or significant (26.5%) increases; large improvements (15.4%) and little improvement (20.4%) were less common, with only 10.6% stating there was no change. As for stress reduction, fairly large numbers reported little change or moderate improvements (30.2%) from their initial condition, while lower percentages registered significant (19.7%), great (8.9%), and substantial (3.6%) improvements. The largest proportion stated that no change at all occurred in their tension level. Of those questioned, 53.6% reported feeling no pressure to wear fragrances or cosmetics for social or cultural reasons. The remainder were divided roughly equally: 17.8% reported feeling under more than just a little pressure, while 28.6% registered an occasional sense that such obligations existed. The majority of participants (64.9%) felt that these products benefited their academic performance, while 29.9% reported no change, and 5.2% stated they had experienced something negative. As far as the beauty conventions 39.7% experienced anxiety that they might not measure up to them while on the contrary 60.3% did not have any worries at all about this issue, 76.1% had anxiety about meeting the standard and more than half reported that their self-esteem (54.4%) was higher when using fragrances or cosmetics than not using them, and only 3.5% stated that it was lower. Most respondents reported that fragrances and cosmetics have a positive effect on their mental state, confidence, and self-esteem.

**TABLE 8:** Mental health and wellbeing, stress and self-perception (n = 461) [n (%)].

Mental health domains		Total
How do you feel after applying fragrances or cosmetics?	More confident	124 (26.9)
	Relaxed and happy	196 (42.5)
	Neutral	89 (19.3)
	Anxious or self-conscious	52 (11.3)
How much do fragrances and cosmetics improve your mood?	No change	49 (10.6)
	Slight improvement	94 (20.4)
	Moderate improvement	125 (27.1)
	Significant improvement	122 (26.5)
	Dramatic improvement	71 (15.4)
How much does using fragrances or cosmetics help reduce stress?	No change	87 (18.9)
	Slight improvement	103 (22.3)
	Moderate improvement	139 (30.2)
	Significant improvement	91 (19.7)
	Dramatic improvement	41 (8.9)
Are you pressured to use fragrances or cosmetics to meet social or cultural expectations?	No	247 (53.6)
	Yes	82 (17.8)
	Sometimes	132 (28.6)
Do you believe using fragrances or cosmetics affects your academic performance by influencing your mood or confidence?	No	138 (29.9)
	Yes positively	299 (64.9)
	Yes negatively	24 (5.2)
Have you ever felt anxious about not meeting beauty standards associated with fragrances or cosmetics?	No	278 (60.3)
	Yes	183 (39.7)
How would you rate your self-esteem when you use fragrances or cosmetics compared to when you do not?	The same	194 (42.1)
	Higher	251 (54.4)
	Lower	16 (3.5)

Supplementary Table S1 presents the results of the bivariate logistic regression analysis (CI: 0.15–0.524, p < 0.001) for predictors of adverse reactions to fragranced and cosmetic products (n = 461).

Age turned out to be a strong indicator, together with 31.0%, 95% CI: 0.182–0.524 odds, supporting the statement that those who were 30–40 years old had a 69.1% lower likelihood of having adverse reactions compared to those who were aged 18–29 years (OR = 0.309, 95% CI). Persons aged 41–50 and those aged 50+ were also significantly less likely than those under 30 years to have this factor negatively affect their reactions (OR = 0.512, 95% CI: 0.287–0.914, p = 0.024; OR = 0.393, 95% CI: 0.170–0.907, p = 0.029, respectively). Marital status was a significant predictor: married respondents had 34.3% lower odds of experiencing adverse reactions than unmarried individuals (OR = 0.343, 95% CI: 0.227–0.520, p < 0.001). The factor was also dependent on the employment situation; the unemployed and retired, in particular, had lower odds of experiencing adverse reactions (OR = 0.229, 95% CI: 0.131–0.401, p < 0.001; OR = 0.190, 95% CI: 0.054–0.669, p = 0.007).

A study has been conducted concerning the use of fragranced products in personal care, household, cleaning and aftershave use which are the main factors that strongly influence the exposure during personal care and to more than twofold (OR = 2.127, 95% CI: 1.329–3.405, p = 0.002) as in the case of aftershave which increases the odds while cleaning supplies, laundry, and household products decrease the odds (OR = 0.582–0.666, p = 0.007–0.038). Exposure to others’ use of laundry and household products also significantly reduced the odds (OR = 0.584 and 0.600, p = 0.006 and 0.009, respectively). Frequency of using fragrances twice a day or four times or more was associated with increased odds of adverse reactions (OR = 1.915, 95% CI: 1.158–3.167, p = 0.011; OR = 1.917, 95% CI: 0.965–3.808, p = 0.024). Cosmetic product use was significant for skincare (OR = 0.386, 95% CI: 0.212–0.704, p = 0.002) and traditional/herbal cosmetics (OR = 0.606, 95% CI: 0.414–0.889, p = 0.010). Monthly cosmetic use was significantly higher than daily (OR = 7.438, 95% CI: 1.555–35.579, p = 0.012). Using 6–10 cosmetic products daily increased the odds (OR = 1.801, 95% CI: 1.004–3.231, p = 0.048). Storage locations, including room cabinets, cars, and handbags, were associated with higher odds of reactions (OR = 1.954–1.979, p = 0.003–0.034). Reading labels or performing allergy tests before using new products was protective, with infrequent allergy testing showing reduced odds (OR = 0.216–0.349, p < 0.001–0.016). In the end, a familial history of allergies significantly increased the risk of adverse reactions (OR = 3.129, 95% CI: 2.108–4.645, p < 0.001), while lack of allergy identification was a barrier (OR = 0.167, 95% CI: 0.072–0.389, p < 0.001). This sheds light on the primary demographic, exposure, and behavioral factors that are significantly associated with negative responses to fragrances and cosmetics.

### 2.9 A multivariable logistic regression model that finds participant adverse reaction predictions

Table 9 presents the conclusive multivariable logistic regression model, which identifies the factors contributing to adverse reactions among participants (n = 461). One of the noteworthy predictors was secondary education, which was associated with a lower likelihood of experiencing adverse reactions (OR = 0.393; 95% CI: 0.201–0.768; p = 0.006), suggesting that individuals with secondary education were less likely to report adverse reactions than their counterparts without secondary education. Moreover, functionalizing cosmetics purchased from stores decreased the risk of adverse reactions and was a predictor (OR = 0.454; 95% CI: 0.208–0.991; p = 0.047). When it comes to safety precautions, testing new products for allergies has been shown to be a very powerful protective factor (OR = 0.108; 95% CI: 0.031–0.378; p = 0.001), with similar protective effects observed on occasionally (OR = 0.284; 95% CI: 0.095–0.849; p = 0.024), seldom (OR = 0.293; 0.099–0.865; p = 0.026), and never (OR = 0.278; 95% CI: 0.099–0.778; p = 0.015).

**TABLE 9:** The final model of predictors of the adverse reactions using multivariable logistic regression (n = 461) [n (%)].

Predictors		OR (95% CI)	P-value
Age group	30–40 years	0.762 (0.325; 1.786)	0.531
	41–50 years	0.688 (0.284; 1.665)	0.407
	Above 50 years	0.616 (0.183; 2.068)	0.433
Education	Secondary	**0.393 (0.201; 0.768)**	0.006*
Occupation	Unemployed	0.768 (0.368; 1.605)	0.483
	Retired	0.619 (0.129; 2.968)	0.548
Marital status	Married	0.502 (0.241; 1.043)	0.065
Exposure to fragranced products (at least once a week) MAQ (by own use)	Cleaning supplies	0.985 (0.562; 1.727)	0.959
	Laundry products	0.709 (0.361; 1.393)	0.319
	Household products	0.598 (0.320; 1.116)	0.106
	Aftershave	1.732 (0.898; 3.340)	0.101
Exposure to fragranced products (at least once a week), MAQ (by others’ use)	Laundry products	0.850 (0.479; 1.509)	0.579
	Household products	1.137 (0.635; 2.036)	0.666
	Aftershave	0.787 (0.467; 1.327)	0.392
Frequency of fragrances usually used in a day	Twice	1.088 (0.491; 2.414)	0.835
	Four times or more	1.186 (0.703; 2.001)	0.522
What categories of cosmetic products do you use? MAQ	Skincare	0.598 (0.243; 1.472)	0.263
	Traditional/herbal cosmetics	0.787 (0.467; 1.327)	0.369
How frequently do you use these cosmetics?	Monthly	5.225 (0.803; 33.999)	0.084
How many cosmetic products do you use daily?	6–10 times	1.834 (0.985; 3.412)	0.056
Where do you purchase cosmetics from? MAQ	Cosmetic shops	**0.454 (0.208; 0.991)**	0.047*
	Online stores	0.870 (0.486; 1.557)	0.640
What is your criterion for selecting cosmetic products? MAQ	Brand reputation	1.398 (0.781; 2.503)	0.259
	Recommendations from others	1.046 (0.587; 1.865)	0.878
	Advertisements	1.227 (0.595; 2.531)	0.579
Where do you store your cosmetics? MAQ	Room cabinets	1.820 (0.791; 4.185)	0.159
	Car	1.023 (0.455; 2.301)	0.957
	Handbags	1.383 (0.789; 2.422)	0.257
Do you read the label or instructions before using cosmetics?	Often	1.841 (0.806; 4.206)	0.147
	Sometimes	1.794 (0.907; 3.550)	0.093
	Rarely	2.061 (0.959; 4.429)	0.064
Do you check the expiry date of cosmetics?	Often	1.615 (0.866; 3.012)	0.131
Do you perform allergy tests before using new products?	Often	**0.108 (0.031; 0.378)**	0.001*
	Sometimes	**0.284 (0.095; 0.849)**	0.024*
	Rarely	**0.293 (0.099; 0.865)**	0.026*
	Never	**0.278 (0.099; 0.778)**	0.015*
How do you feel after applying fragrances or cosmetics?	Relaxed and happy	0.656 (0.370; 1.163)	0.149
	Neutral	0.795 (0.374; 1.690)	0.551
	Anxious or self-conscious	1.305 (0.276; 6.161)	0.737
How much do fragrances and cosmetics improve your mood?	Slight improvement	1.291 (0.600; 2.779)	0.513
Are you pressured to use fragrances or cosmetics to meet social or cultural expectations?	Yes	0.899 (0.471; 1.718)	0.748
Do you believe using fragrances or cosmetics affects your academic performance by influencing your mood or confidence?	Yes positively	**3.776 (2.082; 6.848)**	0.001*
	Yes negatively	2.120 (0.614; 7.315)	0.234
Do any of your family members have a history of allergies?	Yes	1.509 (0.884; 2.577)	0.132
Do you have any allergies to	No known allergies	**0.358 (0.204; 0.629)**	0.001*

Bold values indicate statistically significant associations (*p* < 0.05) in the multivariate logistic regression model.

In the end, participants who identified the use of fragrances or cosmetics as advantageous for university performance had increased odds of experiencing an adverse reaction (OR = 3.776; 95% CI: 2.082–6.848; p = 0.001); however, no known allergies had it even unlikely to experiencing an adverse reaction (OR = 0.358, 95% CI: 0.204–0.629; p = 0.001).

## 3 Discussion

This study presents findings on the use of cosmetics and fragrances among adults in the United Arab Emirates. Our results show that among young females, the proportion of product use is very high, specifically in skincare and haircare products. This trend, of course, closely parallels various demographic patterns seen globally (Mohammed, 2023; Naveed, 2014; Steinemann and Goodman, 2019). Thus, we see that people are affected by quite different cultures, social norms, and job-market dynamics worldwide at this stage. Relatively close to one another are the regions where Hart et al. (2020) and Olcer et al. (2023) discussed it. Among participants in our study who had received a university education, greater exposure to a globalized vision of beauty and the latest cosmetic fashions may imply greater variety and more frequent use. Fragrance exposure was virtually universal in our sample, with personal care and household products, especially air fresheners or deodorizers, being the most common sources. There was also a lot of indirect exposure to other people’s use; fragrance chemicals are thoroughly mixed into the atmosphere of places where people work together and live with their families. Our findings align well with research findings from the West, where more than 85% of people in the general population report often coming into contact with fragranced products. Often, the researchers have discovered, this results in the indoor environment serving as a site of continuous chemical insult (Rádis-Baptista, 2023). In indoor spaces filled with volatile organic compounds (VOCs) and other fragrance chemicals, light absorbed by the human skin may trigger a reaction even after years of exposure (Račić et al., 2025).

The high frequency of reported adverse reactions in our study was undeniable: 35.4% of participants reported symptoms affecting the skin, respiratory system, and other organs. The most common dermatological side effects were redness, rashes, itching, and flaking, which are nearly identical to the findings from a cross-sectional study in India (Abiramy et al., 2025). Among the different reactions, respiratory symptoms, headaches, and mucous membrane irritation were closely associated with air freshener use, thereby continuing the previously established connection between fragrance exposure, airway irritation and neurological effects (Ahmed and Mirza, 2020; Al-Shebel and Al-Sowayan, 2025).

While serious systemic episodes were rare, the high proportion of skin problems prompted preventive measures. The public should also be educated about product safety. Regarding knowledge, attitudes, and practices (KAP) about fragrance, a moderate level of awareness of chemical hazards was observed; fewer than 50% recognized that fragranced products are complex mixtures of chemicals, and only 11.5% knew that all fragrance ingredients should be disclosed. Most people thought natural ingredients were inherently safer for personal care, perpetuating a common misconception in previous surveys (Lunny et al., 2017; Trifunovski, 2024). Affective elements, such as the role of fragrance in enhancing one’s appearance or improving mood, elicited strong support and positive feelings. More than half of the participants reported that, after applying perfumes and cosmetics, they felt emotionally uplifted, had improved self-esteem, and reduced stress. Such psychosocial benefits are supported by recent studies showing that the use of cosmetics and perfumes can indeed increase self-confidence, particularly among females and young individuals (Croijmans et al., 2021; Fares et al., 2019).

In this study, cosmetics were used daily in a variety of product forms and for a range of purposes, predominantly for skin care, hair care, personal care items, and makeup. Friends or family members may also borrow cosmetics, which was quite common among students. This practice increases the risk of cross-infection. Purchases were made at cosmetic shops, pharmacies, and online platforms, a combination of convenient locations and personal preferences for brands known for their quality. Monthly spending on personal care was high, consistent with international trends among upper-income groups (Newton et al., 2024).

Educational campaigners trained activists on preventive safety measures, safe storage standards, and potential risks associated with using multiple products per occurrence. The importance of public safety education campaigns addressing these issues was also underlined in the present study.

Fragrances and cosmetics were singled out as the most preferred products by a large margin. The participants reported feeling comfortable and experiencing emotional release. Such boosts to self-identity are conducive to daily functioning and social security. Based on our multivariate analysis, product users who perceived benefits for academic achievement had an even higher risk of adverse reactions (OR = 3.562). This phenomenon suggests that individuals who frequently use products to enhance their appearance or improve performance may inadvertently also increase their exposure to sensitizers and irritants with which they come into contact. In this connection, Kaushik et al. (2023) found that heavy users of everyday cosmetics had cumulative rates almost four times higher than those found in similar assessments decades ago.

Multivariable regression analysis also identified protective and risk factors. For those involved in allergy testing, the odds of adverse reactions were considerably lower phenomenon known as patch testing (Azam, 2024; Ophaug and Schwarzenberger, 2020). By contrast, people with higher educational attainment were more likely to react, a finding that may reflect greater exposure to multiple products, trend-driven cosmetics, and overconfidence. Participants without prior allergies were considerably less likely to report any side effects, highlighting the role of pre-existing skin conditions in the development of this sensitivity (Mehta et al., 2024). The surprising observation that reading product labels was associated with a higher probability of an adverse reaction could be a case of reverse causality, in which individuals who have experienced a reaction are more likely to be vigilant about reading labels. This issue has been highlighted in customer safety surveys (Abiramy et al., 2025).

In summary, the above findings reveal a paradoxical aspect of beauty products and perfumes, their use can lead to positive bodily and psychological effects as well as to dermatological problems, mood suppression, and even health issues for the body if proper maintenance steps are not taken by users, through inhalation, absorption via rubbing into the skin or under the fingernails, and even systemic health problems. In 2025, the United Arab Emirates Cabinet introduced a revised national regulation applicable to the sale, importation, and production of cosmetics and personal care products. This regime guarantees safety, quality, and compliance with international standards before a product can enter or remain in the market. The scheme covers the local manufacturing, import, distribution, and sale of cosmetics and personal care products; all such products must be registered and certified before being marketed in the UAE. The registration process includes safety, quality testing, and accurate labeling in both Arabic and English, which is align with the necessarity to develop UAE-specific public health intervention programs where different methods like educating consumers about safe use, doing allergy tests, and making people aware of the harm caused by cumulative exposure are needed with regulatory supervision, and enforcement of good practice that assures correct labeling and disclosure of all ingredients. As most research in this region has relied on Western studies, there is a significant lack of region-specific knowledge in the Middle East. These results help address this gap by providing a basis for longitudinal studies on long-term effects, interactions among different products, and mental health symptoms associated with these applications.

### 3.1 Limitations

This is a cross-sectional study, which means it cannot be used to make causal inferences; thus, correlations between reported adverse reactions and the medicines taken may be mere associations rather than causal relationships. Faulty patient reports may also have occurred due to a lack of understanding. Moreover, because the data were self-reported, there is a potential for bias in the recall of matters, as people’s tendency to give answers that they think others want to hear, such as how much of a product they have used, or in describing symptoms, is another social bias. Employees comprised the majority of participants, including highly educated university students and other employees. This makes it challenging to apply the results to other sectors of society, including both sexes and individuals with lower levels of education. The estimates of exposure were derived from questions on frequency and self-reported consumption, using surrogate variables rather than directly measuring consumption. This could have led to an under- or overestimation of actual exposure levels at times. Finally, the study did not examine the long-term health effects of chronic, multiple product exposure or cumulative effects.

### 3.2 Conclusion

The study examines the use of cosmetics and fragrances among adult females in the UAE, encompassing not only widespread exposure but also their health effects, as one of its main themes, young adults and students have been reported as the heaviest daily users of various products, mainly skincare, haircare, and fragrances, which in most cases lead to dermatological, respiratory, and mild systemic reactions. While perfumes and makeup have the power to transform negative feelings into positive ones in the psychosocial realm, such as improved mood, confidence, and self-esteem, the regular use of many items and a lack of preventive measures increase the risk of health problems. Nonetheless, the general public has a limited understanding of the dangers posed by the chemicals involved, and the practice of allergy testing, which serves as a protective measure, is not widespread. In addition, this research lays the foundation for future longitudinal studies to assess health impacts and exposure patterns in the Middle East, particularly in the UAE, where several key regulations, standards, and initiatives governing cosmetics and fragrance products align with these recommendations. The Emirates Conformity Assessment Scheme (ECAS), which is a compulsory registration system for cosmetic and perfumery products that makes it compulsory for them to complete and submit for examination and certification before the item is imported or sold, products that do not have an ECAS certificate can be seized or rejected by customs, complies with clearly stated safety, labeling, and packaging technical requirements (Store, 2024).

These recommendations align with WHO guidance on indoor air pollutants and chemical exposures (World Health Organization, 2010) and with regional requirements on cosmetics safety and labeling (GCC Standardization Organization, 2024).

## Data Availability

The original contributions presented in the study are included in the article/Supplementary Material, further inquiries can be directed to the corresponding author.

## References

[B1] Abiramy P. Maharani B. Saritha M. Mathiyalagen P. Balagurumoorthy M. Mohanan S. (2025). The pattern of use of cosmetics and awareness of cosmetovigilance among medical students in Puducherry: a cross-sectional study. Cureus 17 (2), e78335. 10.7759/cureus.78335 40034636 PMC11874450

[B2] Ahmed F. Mirza F. (2020). Fragrances and their effects on public health: a narrative literature review. Environmental Health Association of Quebec: Quebec, QC, Canada.

[B3] Air Quality, Energy and Health (AQE) (2010). WHO guidelines for indoor air quality: selected pollutants. Available online at: https://www.who.int/publications/i/item/9789289002134?utm_source (Accessed February 4, 2026). 23741784

[B4] Al-Shebel D. K. Al-Sowayan N. S. (2025). Investigating the impact of aromatic scents on vital systems. Adv. Biosci. Biotechnol. 16 (5), 178–189. 10.4236/abb.2025.165011

[B5] Alblooshi S. (2025). The impact of perfumes and cosmetic products on human health: a narrative review. Front. Toxicol. 7, 1646075. 10.3389/ftox.2025.1646075 40949028 PMC12425936

[B6] Alosyli F. Aljebrin L. Alnowaiser N. Alodhilah Y. Ibrahim N. Anaam M. S. (2024). Insight of saudi users of cosmetic regarding cosmetovigilance: a survey of knowledge, attitude and practice. Open Public Health J. 17 (1), e18749445350785. 10.2174/0118749445350785241028071257

[B7] Athar M. (2020). Detrimental effects of perfumes, aroma and cosmetics. J. Dermatol Cosmetol. 4, 42–44. 10.15406/jdc.2020.04.00149

[B8] Azam U. (2024). A new era in cosmetology. Eur. J. Biomed. 11 (5), 91–102.

[B9] Borowska S. Brzóska M. M. (2015). Metals in cosmetics: implications for human health. J. Applied Toxicology 35 (6), 551–572. 10.1002/jat.3129 25809475

[B10] Croijmans I. Beetsma D. Aarts H. Gortemaker I. Smeets M. (2021). The role of fragrance and self-esteem in perception of body odors and impressions of others. PloS One 16 (11), e0258773. 10.1371/journal.pone.0258773 34780484 PMC8592444

[B11] Fares K. Hallit S. Haddad C. Akel M. Khachan T. Obeid S. (2019). Relationship between cosmetics use, self-esteem, and self-perceived attractiveness among Lebanese women. J. Cosmetic Science 70 (1), 47–56. 30856095

[B12] Getachew M. Tewelde T. (2018). Cosmetic use and its adverse events among female employees of jimma university, southwest Ethiopia. Ethiop. J. Health Sciences 28 (6), 717–724. 10.4314/ejhs.v28i6.6 30607088 PMC6308756

[B13] Hart L. B. Walker J. Beckingham B. Shelley A. Alten Flagg M. Wischusen K. (2020). A characterization of personal care product use among undergraduate female college students in South Carolina, USA. J. Exposure Science and Environmental Epidemiology 30 (1), 97–106. 10.1038/s41370-019-0170-1 31548624

[B14] Huang P.-C. Liao K.-W. Chang J.-W. Chan S.-H. Lee C.-C. (2018). Characterization of phthalates exposure and risk for cosmetics and perfume sales clerks. Environ. Pollut. 233, 577–587. 10.1016/j.envpol.2017.10.079 29102888

[B15] In J. (2017). Introduction of a pilot study. Korean Journal Anesthesiology 70 (6), 601–605. 10.4097/kjae.2017.70.6.601 29225742 PMC5716817

[B16] Kaushik M. Farooq U. Ali M. S. Ansari M. J. Iqbal Z. Mirza M. A. (2023). Safety concern and regulatory status of chemicals used in cosmetics and personal care products. Dermato 3 (2), 131–157. 10.3390/dermato3020011

[B17] Klaschka U. (2020). “This perfume makes me sick, but I like it.” representative survey on health effects associated with fragrances. Environ. Sci. Eur. 32 (1), 30. 10.1186/s12302-020-00311-y

[B18] Lucca J. M. Joseph R. Al Kubaish Z. H. Al-Maskeen S. M. Alokaili Z. A. (2020). An observational study on adverse reactions of cosmetics: the need of practice the cosmetovigilance system. Saudi Pharm. J. 28 (6), 746–753. 10.1016/j.jsps.2020.04.017 32550807 PMC7292860

[B19] Lunny S. Nelson R. Steinemann A. (2017). Something in the air but not on the label: a call for increased regulatory ingredient disclosure for fragranced consumer products. UNSWLJ 40, 1366. 10.53637/fzxh4269

[B20] Mehta G. Tyagi D. R. Sachdeva M. Tripathi R. Tyagi H. (2024). An observational study on cosmetics use-related adverse effects: cosmetovigilance need of the day. Drug Res. 74 (04), 164–170. 10.1055/a-2251-6655 38467158

[B21] Mohammed A. H. Hassan B. A. R. Wayyes A. M. Al-Tukmagi H. F. Blebil A. Dujaili J. (2023). Exploring the quality of life of cosmetic users: a cross-sectional analysis from eight Arab countries in the Middle East. J. Cosmet. Dermatology 22 (?), 296–305. 10.1111/jocd.15085 35567513 PMC10286760

[B22] Naveed N. (2014). The perils of cosmetics. J. Pharmaceutical Sciences Research 6 (10), 338.

[B23] Newton J. Ochoa L. Reinschmidt A. Vassar J. Wellman A. Vargas M. (2024). Is beauty worth the risk? self-Confidence is the key motivating factor driving tanning bed use among undergraduate students at South Dakota universities. Int. J. Women's Dermatology 10 (2), e128. 10.1097/JW9.0000000000000128 38572264 PMC10986912

[B24] Odencrantz A. Offenberg D. (2020). Beyond a tube of red lipstick: an economic valuation of estee lauder and the beauty industry.

[B25] Olcer Z. Cal A. Unal N. Oztas B. Oge G. (2023). Examining the use of cosmetic products and the awareness of healthy life among university students. Turkish J. Dermatology 17 (3), 79–87. 10.4103/tjd.tjd_136_22

[B26] Ophaug S. Schwarzenberger K. (2020). Pitfalls in patch testing: minimizing the risk of avoidable false-negative reactions. Dermatol. Clin. 38 (3), 293–300. 10.1016/j.det.2020.02.007 32475507

[B28] Panico A. Serio F. Bagordo F. Grassi T. Idolo A. De Giorgi M. (2019). Skin safety and health prevention: an overview of chemicals in cosmetic products. J. Preventive Medicine Hygiene 60 (1), E50. 10.15167/2421-4248/jpmh2019.60.1.1080 31041411 PMC6477564

[B29] Račić N. Terzić I. Karlović N. Bošnjaković A. Terzić T. Jakovljević I. (2025). Volatile organic compounds (VOCs) and polycyclic aromatic hydrocarbons (PAHs) in indoor environments: a review and analysis of measured concentrations in Europe. Indoor Air 2025 (1), 5945455. 10.1155/ina/5945455

[B30] Rádis-Baptista G. (2023). Do synthetic fragrances in personal care and household products impact indoor air quality and pose health risks? J. Xenobiotics 13 (1), 121–131. 10.3390/jox13010010 36976159 PMC10051690

[B31] Sealey L. Hughes B. Sriskanda A. Guest J. Gibson A. Johnson-Williams L. (2016). Environmental factors in the development of autism spectrum disorders. Environ. Int. 88, 288–298. 10.1016/j.envint.2015.12.021 26826339

[B32] Shaaban H. Alhajri W. (2020). Usage patterns of cosmetic and personal care products among female population in Saudi Arabia: important factors for exposure and risk assessment. J. Environmental Public Health 2020 (1), 8434508. 10.1155/2020/8434508 32322284 PMC7168713

[B33] Sowndhararajan K. Kim S. (2016). Influence of fragrances on human psychophysiological activity: with special reference to human electroencephalographic response. Sci. Pharmaceutica 84 (4), 724–752. 10.3390/scipharm84040724 27916830 PMC5198031

[B34] Steinemann A. (2018). Exposures and effects from fragranced consumer products in Sweden. Air Qual. Atmos. and Health 11 (5), 485–491. 10.1007/s11869-018-0565-5 PMC5093181 27867426

[B35] Steinemann A. Goodman N. (2019). Fragranced consumer products and effects on asthmatics: an international population-based study. Air Qual. Atmos. and Health 12 (6), 643–649. 10.1007/s11869-019-00693-w

[B36] Store, G. S. (2024). Gso 1943:2024 - standards Store - GCC standardization organization. Riyadh, Saudi Arabia: GCC Standardization Organization.

[B37] Trifunovski A. (2024). Examining women’s knowledge, health risk perceptions, beliefs and behaviours in relation to toxic chemicals commonly found in personal care and household products: a mixed-methods approach 10.3390/toxics13050414 PMC12116110 40423493

