# Supplementary material for: From beauty to burden: mapping the health and psychosocial impacts of fragrances and cosmetic products use in the UAE
Source: Front Toxicol. 2026 Apr 15;8:1758512. doi: 10.3389/ftox.2026.1758512 (PMC13124122; doi:10.3389/ftox.2026.1758512)
Supplement: Supplementary file 1 [file Table1.docx]

**Supplementary Table S1. The predictors of adverse reactions by the bivariable logistic regression (n=461) [n (%)]**

| **Predictors** | | **OR (95% CI)** | **P-value** |
| --- | --- | --- | --- |
| Age Group  [Ref: 18-29 years] | 30–40 years | 0.309 (0.182; 0.524) | **˂0.001*** |
|  | 41–50 years | 0.512 (0.287; 0.914) | **0.024*** |
|  | Above 50 years | 0.393 (0.170; 0.907) | **0.029*** |
| Education [Ref: lower than secondary] | Secondary | 0.116 (0.021; 0.638) | **0.013*** |
|  | University | 0.260 (0.050; 1.359) | 0.110 |
|  | Postgraduate | 0.283 (0.049; 1.636) | 0.159 |
| Occupation [Ref: Student] | Employed | 0.687 (0.440; 1.074) | 0.100 |
|  | Unemployed | 0.229 (0.131; 0.401) | **˂0.001*** |
|  | Retired | 0.190 (0.054; 0.669) | **0.007*** |
| Marital Status [Ref: Single] | Married | 0.343 (0.227; 0.520) | **˂0.001*** |
|  | Divorced/widowed | 0.974 (0.318;2.981) | 0.964 |
| Exposure to fragranced products (at least once a week) MAQ (By own use)  [Ref: No] for each option | Air Fresheners and Deodorizers | 1.139 (0.777; 1.670) | 0.504 |
|  | Personal Care products | 0.721 (0.389; 1.335) | 0.298 |
|  | Cleaning Supplies | 0.582 (0.392; 0.863) | **0.007*** |
|  | Laundry Products | 0.610 (0.406; 0.918) | **0.018*** |
|  | Household Products | 0.666 (0.454; 0.978) | **0.038*** |
|  | Aftershave | 2.127 (1.329; 3.405) | **0.002*** |
| Exposure to fragranced products (at least once a week) MAQ (By others use) [Ref: No] for each option | Air Freshener and deodorizers | 0.718 (0.478; 1.078) | 0.110 |
|  | Personal Care products | 0.693 (0.468; 1.025) | 0.066 |
|  | Cleaning Supplies | 0.829 (0.559; 1.230) | 0.352 |
|  | Laundry Products | 0.584 (0.398; 0.858) | **0.006*** |
|  | Household Products | 0.600 (0.408; 0.882) | **0.009*** |
|  | Aftershave | 0.531 (0.355; 1.739) | **0.002*** |
| Frequency of using Fragrance  [Ref: daily] | Weekly | 1.871 (0.820; 4.268) | 0.137 |
|  | Monthly | 5.612 (0.579; 4.433) | 0.138 |
|  | For special occasions | 1.497 (0.396; 5.658) | 0.552 |
| Number of Fragrance Products used Daily [Ref: 1-2 times] | 3-5 times | 1.385 (0.920; 2.085) | 0.119 |
|  | 6-10 times | 1.614 (0.739; 3.528) | 0.230 |
|  | More than 10 | 1.206 (0.481; 3.024) | 0.689 |
| Frequency of fragrances usually used in a day [Ref: Once] | Twice | 1.915 (1.158; 3.167) | **0.011*** |
|  | Thrice | 1.513 (0.880; 2.599) | 0.134 |
|  | Four times or more | 1.917 (0.965; 3.808) | **0.024*** |
| Money spent on Fragrance monthly AED [Ref: less than 100] | 100-200 | 1.333 (0.585; 3.038) | 0.494 |
|  | 200-500 | 0.965 (0.464; 2.010) | 0.925 |
|  | More than 500 | 0.924 (0.446; 1.914) | 0.832 |
| What categories of cosmetic products do you use? MAQ  [Ref: No] for each option | Skincare | 0.386 (0.212; 0.704) | **0.002*** |
|  | Haircare | 0.577 (0.326; 1.022) | 0.059 |
|  | Makeup | 0.801 (0.491; 1.309) | 0.377 |
|  | Perfumes | 0.835 (0.539; 1.296) | 0.422 |
|  | Personal care | 0.641 (0.389; 1.058) | 0.082 |
|  | Nail care | 0.953 (0.639; 1.422) | 0.814 |
|  | Traditional/herbal cosmetics | 0.606 (0.414; 0.889) | **0.010*** |
| How frequently do you use these cosmetics? [Ref: daily] | Weekly | 1.051 (0.619; 1.784) | 0.854 |
|  | Monthly | 7.438 (1.555; 35.579) | **0.012*** |
|  | Special occasions | 0.775 (0.267; 2.251) | 0.639 |
| How many cosmetic products do you use daily? [Ref: 1-2 times] | 3-5 times | 0.848 (0.523; 1.376) | 0.505 |
|  | 6-10 times | 1.801 (1.004; 3.231) | **0.048*** |
|  | More than 10 | 1.146 (0.566; 2.320) | 0.705 |
| How frequently do you usually use cosmetics in a day? [Ref: Once] | Twice | 0.981 (0.630; 1.526) | 0.931 |
|  | Thrice | 1.387 (0.761; 2.528) | 0.286 |
|  | Four times or more | 1.206 (0.580; 2.506) | 0.616 |
| Do you share cosmetics with others (e.g., friends, family members)?  [Ref: Frequently] | Often | 0.416 (0.153; 1.130) | 0.085 |
|  | Sometimes | 0.470 (0.203; 1.085) | 0.077 |
|  | Rarely | 0.501 (0.220; 1.140) | 0.099 |
|  | Never | 0.629 (0.269; 1.472) | 0.285 |
| Where do you purchase cosmetics from? MAQ [Ref: No] for each option | Pharmacy | 1.361 (0.909; 2.037) | 0.135 |
|  | Supermarkets/ hypermarkets | 0.735 (0.500; 1.080) | 0.117 |
|  | Cosmetic shops | 0.412 (0.234; 0.726) | **0.002*** |
|  | Online stores | 1.714 (1.159; 2.534) | **0.007*** |
| What is your criteria for selecting cosmetic products? MAQ  [Ref: No] for each option | Brand reputation | 1.804 (1.215; 2.680) | **0.003*** |
|  | Recommendations from others | 1.647 (1.115; 2.433) | **0.012*** |
|  | Advertisements | 1.773 (1.034; 3.040) | **0.037*** |
|  | Price / Cost | 1.212 (0.816; 1.801) | 0.340 |
|  | Quality | 0.892 (0.592; 1.343) | 0.583 |
| Where do you store your cosmetics? MAQ  [Ref: No] for each option | Room cabinets | 1.979 (1.182; 3.313) | **0.009*** |
|  | Bathroom | 0.963 (0.658; 1.408) | 0.844 |
|  | Car | 1.954 (1.053; 3.626) | **0.034*** |
|  | Handbags | 1.824 (1.223; 2.721) | **0.003*** |
|  | Fridge | 1.336 (0.799; 2.235) | 0.270 |
| How much do you spend on cosmetics per month? AED [Ref: less than 100] | 100-200 | 1.950 (0.838; 4.537) | 0.121 |
|  | 200-500 | 1.723 (0.780; 3.809) | 0.179 |
|  | More than 500 | 1.043 (0.466; 2.339) | 0.918 |
| Do you read the label or instructions before using cosmetics?  [Ref: Frequently] | often | 1.929 (1.013; 3.672) | **0.046*** |
|  | sometimes | 1.724 (1.001; 2.969) | **0.050*** |
|  | rarely | 2.006 (1.095; 3.673) | **0.024*** |
|  | never | 0.643 (0.283; 1.459) | 0.290 |
| Do you check the expiry date of cosmetics? [Ref: Frequently] | often | 1.202 (1.288; 3.760) | **0.004*** |
|  | sometimes | 1.324 (0.780; 2.249) | 0.299 |
|  | rarely | 1.553 (0.882; 2.733) | 0.127 |
|  | never | 1.024 (0.453; 2.314) | 0.955 |
| Do you perform allergy tests before using new products?  [Ref: Frequently] | often | 0.229 (0.084; 0.625) | **0.004*** |
|  | sometimes | 0.216 (0.092; 0.508) | **˂0.001*** |
|  | rarely | 0.349 (0.148; 0.819) | **0.016*** |
|  | never | 0.225 (0.100; 0.507) | **˂0.001*** |
| How do you feel after applying fragrances or cosmetics?  [Ref: more confident] | relaxed and happy | 0.593 (0.375; 0.936) | **0.025*** |
|  | neutral | 0.570 (0.325; 0.999) | **0.049*** |
|  | anxious or self-conscious | 0.166 (0.069; 0.396) | **˂0.001*** |
| How much do fragrances and cosmetics improve your mood?  [Ref: no change] | slight improvement | 0.407 (0.194; 0.854) | **0.017*** |
|  | moderate improvement | 1.154 (0.593; 2.247) | 0.673 |
|  | significant improvement | 0.650 (0.329; 1.284) | 0.215 |
|  | dramatic improvement | 0.818 (0.390; 1.717) | 0.596 |
| How much does using fragrances or cosmetics help reduce stress?  [Ref: no change] | slight improvement | 1.132 (0.618; 2.074) | 0.688 |
|  | moderate improvement | 1.648 (0.941; 2.887) | 0.081 |
|  | significant improvement | 0.889 (0.471; 1.679) | 0.717 |
|  | dramatic improvement | 1.349 (0.623; 2.919) | 0.448 |
| Are you pressured to use fragrances or cosmetics to meet social or cultural expectations? [Ref: No] | yes | 2.288 (1.377; 3.803) | **0.001*** |
|  | sometimes | 0.890 (0.566; 1.401) | 0.615 |
| Do you believe using fragrances or cosmetics affects your academic performance by influencing your mood or confidence? [Ref: No] | yes positively | 2.315 (1.465; 3.658) | **˂0.001*** |
|  | yes negatively | 3.915 (1.600; 9.581) | **0.003*** |
| Have you ever felt anxious about not meeting beauty standards associated with fragrances or cosmetics? [Ref: No] | yes | 1.381 (0.939; 2.032) | 0.101 |
| How would you rate your self-esteem when you use fragrances or cosmetics compared to when you do not? [Ref: The same] | higher | 1.250 (0.845; 1.849) | 0.264 |
|  | lower | 1.191 (0.415; 3.420) | 0.746 |
| Does any of your family members have a history of allergies? [Ref: No] | yes | 3.129 (2.108; 4.645) | **˂0.001*** |
| Do you have any allergies to:  [Ref: Medication] | food | 0.667 (0.250; 1.776) | 0.417 |
|  | environmental factors | 0.571 (0.232; 1.407) | 0.223 |
|  | no known allergies | 0.167 (0.072; 0.389) | **˂0.001*** |

**^Abbreviations: Ref: reference, OR: odds ratio, CI: confidence interval. Variables with a^*^p^*^-value less than 0.05 in the bivariable logistic regression analysis were included in the multivariable logistic regression. Significant values are bold. Experience any adverse reaction categories (yes and no) as a binary variable with the values “0” and “1” were entered into the regression model as a dependent variable.^**
